# Fibrin and Fibrinolytic Enzyme Cascade in Thrombosis: Unravelling the Role

**DOI:** 10.3390/life13112196

**Published:** 2023-11-11

**Authors:** Rajni Singh, Prerna Gautam, Chhavi Sharma, Alexander Osmolovskiy

**Affiliations:** 1 Amity Institute of Microbial Technology, Amity University Uttar Pradesh, Noida 201301, India; prerna.gautam.prerna@gmail.com (P.G.); sharmachhavi27@gmail.com (C.S.); 2 Biological Faculty of Lomonosov, Moscow State University, 119234 Moscow, Russia

**Keywords:** fibrin, fibrinolysis, fibrinolytic assays, thrombosis, microbial fibrinolytic enzymes

## Abstract

Blood clot formation in blood vessels (thrombosis) is a major cause of life-threatening cardiovascular diseases. These clots are formed by αA-, βB-, and ϒ-peptide chains of fibrinogen joined together by isopeptide bonds with the help of blood coagulation factor XIIIa. These clot structures are altered by various factors such as thrombin, platelets, transglutaminase, DNA, histones, and red blood cells. Various factors are used to dissolve the blood clot, such as anticoagulant agents, antiplatelets drugs, fibrinolytic enzymes, and surgical operations. Fibrinolytic enzymes are produced by microorganisms (bacteria, fungi, etc.): streptokinase of *Streptococcus hemolyticus,* nattokinase of *Bacillus subtilis* YF 38, bafibrinase of *Bacillus* sp. AS-S20-I, longolytin of *Arthrobotrys longa*, versiase of *Aspergillus versicolor* ZLH-1, etc. They act as a thrombolytic agent by either enhancing the production of plasminogen activators (tissue or urokinase types), which convert inactive plasminogen to active plasmin, or acting as plasmin-like proteins themselves, forming fibrin degradation products which cause normal blood flow again in blood vessels. Fibrinolytic enzymes may be classified in two groups, as serine proteases and metalloproteases, based on their catalytic properties, consisting of a catalytic triad responsible for their fibrinolytic activity having different physiochemical properties (such as molecular weight, pH, and temperature). The analysis of fibrinolysis helps to detect hyperfibrinolysis (menorrhagia, renal failure, etc.) and hypofibrinolysis (diabetes, obesity, etc.) with the help of various fibrinolytic assays such as a fibrin plate assay, fibrin microplate assay, the viscoelastic method, etc. These fibrinolytic activities serve as a key aspect in the recognition of numerous cardiovascular diseases and can be easily produced on a large scale with a short generation time by microbes and are less expensive.

## 1. Introduction

Cardiovascular diseases usually refer to conditions that involve the narrowing or blocking blood vessels, leading to stroke, heart attack, and angina [1]. The report provided by the World Health Organisation has shown that every year 17 million people die due to cardiovascular disorders. One of the main reasons for cardiovascular diseases is intravascular thrombosis (formation of blood clots in blood vessels) [2], which is caused due to the excessive activation of the blood coagulation cascade, wherein factor XII (the serine protease used to start the coagulation cascade) plays a role in thrombosis [3]. The high rates of mortality and morbidity are affiliated with venous and arterial thromboembolism (blockage of a blood vessel by a blood clot that has been dislodged from another site in the circulation) [4]. These blood clots are composed of fibrin fibres which provide a three-dimensional protein network and elasticity. These clots can be dissolved by the hydrolysis of fibrin by plasmin protein via the fibrinolysis process [2]. Fibrinolysis exhibits two kinds of activities: increased fibrinolysis that is seen in menorrhagia, renal failure, cirrhosis, malignancies, leukaemia, etc., and decreased fibrinolysis as seen in diabetes, obesity, hyperlipidaemia, and atherosclerosis [5].

**Figure 1:**
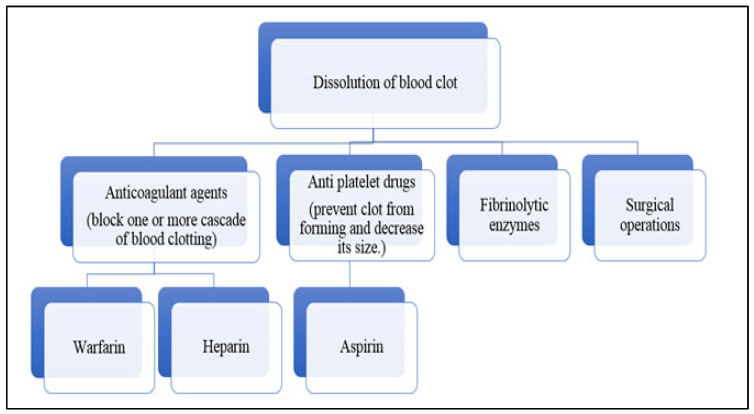
Different therapies for blood clot dissolution.

Nowadays, various enzymes are used as anti-inflammatories, anticoagulants, oncolytics, thrombolytics, antimicrobials, digestive aids, and mucolytics [6]; among them, the enzymes responsible for clot dissolution are anticoagulant agents, antiplatelet drugs, and fibrinolytic enzymes (Figure 1) [7]). Fibrinolytic enzymes are extracted from microbes acting as potent biochemical catalysts with various biochemical applications. These fibrinolytic enzymes like streptokinase [8], nattokinase, longolytin, etc., show thrombolytic properties to treat cardiovascular diseases [9].

The fibrinolytic activity of these enzymes is estimated using various methods such as a fibrin plate assay, fibrin microplate assay, viscoelastic method, euglobulin clot lysis time, global fibrinolytic capacity, and rapid fibrin plate assay.

## 2. Materials and Methods

We performed an extensive literature search using scientific databases and commercial search engines like PubMed, Web of Science, ScienceDirect, ResearchGate, Google Scholar, etc., to search research papers, articles, and books for information related to mechanisms and applications. In total, 1896 different articles were scrutinized on the basis of availability; 53 elements of the latest data were finally selected with cross references, and information was compiled for the present study.

## 3. Results and Discussion

Worldwide cardiovascular diseases (CVDs) remain the biggest cause of death, accounting for about 31% of the total mortality rate (17.9 million deaths per year), which could exceed 23.6 million by 2030. A study from the Global Burden of Disease estimated a CVD death rate of 235 per 100,000 globally and estimated that 10.9 million people will be hospitalized for cardiovascular diseases by 2023, with 2.1 million deaths [2,3].

### 3.1. Cardiovascular Diseases and Thrombosis

Blood clotting and dissolution (i.e., fibrin formation and fibrinolysis) act in equilibrium. An imbalance in this equilibrium results in intravascular blood clotting, limiting the flow of blood through veins and arteries, resulting in thrombosis, leading to cardiac ailments. The leading cause of death worldwide is cardiovascular diseases which can be caused due to dysfunctional endothelial cells of the blood vessels. This leads to inflammation in the blood vessels and atherosclerotic lesions, resulting in myocardial infarction and stroke [10]. Cardiovascular diseases influenced by fibrinolytic activity occur in several other thrombotic disease states, such as acute myocardial infarction, coronary artery disease, venous thromboembolism, sepsis, and insulin-resistance syndrome. Such severe cases are associated with abnormalities such as obesity, overweight, diabetes mellitus, metabolic syndrome, hypertension, and lipid disorders (decreased HDL cholesterol and elevated triglyceride levels) [11,12,13,14]. Many studies have shown a strong relationship between plasma fibrinogen, incidence of stroke, and ischemic heart diseases. In the onset of ischemic heart diseases, factor VII (proconvertin, which causes blood to clot), and extrinsic pathway activity play a crucial role, whereas lipoproteins have a major impact on coagulability and atherogenesis [15,16]. 

Thrombosis is the development of blood clots inside the blood vessel, blocking the blood flow of the circulatory system. There can be two types: arterial thrombosis, which is concerned with high shear stress forming white thrombi (platelet-rich), and antiplatelet drugs like aspirin, triflusal, etc., are required for its treatment, and venous thrombosis, which concerns with low shear stress and blood flow forming red thrombi (red blood cell-rich) and anticoagulant drugs like heparin, warfarin, etc., are required for its treatment [11]. Three features that contribute to venous thrombosis (the Virchow Triad) are stasis, hypercoagulability, and endothelial injury [17]. A serious complication of thrombosis is seen in patients affected by myeloid and lymphoid leukaemia as leukemic cells activate procoagulants of platelets initiating thrombosis [18]. Procoagulant platelets facilitate the assembly of prothrombinase and tenase and thus result in thrombin burst, fibrin formation, and aggregation to create a fragile fibrin network [19] Deep venous thrombosis has a high risk for repeated venous thromboembolism that prevail for many years [20]. Oral anticoagulants are the standard treatment for deep vein thrombosis. It is also treated by drugs such as urokinase, streptokinase, tissue plasminogen activator, etc., which are injected into the foot/arm or directly at the site of the blood clot to dissolve the blood clot [21].

Ongoing clinical trials are determining the clinical efficiency of anti-inflammatory therapy in reducing the thrombus and defining the role of anti-platelet and anti-thrombotic therapy in inflammatory states [22].

### 3.2. Molecular Mechanism of Clot Formation

The basic unit of blood clots is fibrinogen, 340 kDa trinodular protein, present at a high concentration (2–4 mg/mL) in plasma and secreted by hepatocytes. It is composed of a dimer where an individual subunit consists of three polypeptide chains cross-linked by 29 disulfide linkages (αA-, βB- and Υ-chains) bound together as thread-like structures [23,24]. It participates in many biological processes like wound healing, haemostasis, inflammation, atherosclerosis, thrombosis, angiogenesis, etc. [25].

**Figure 2:**
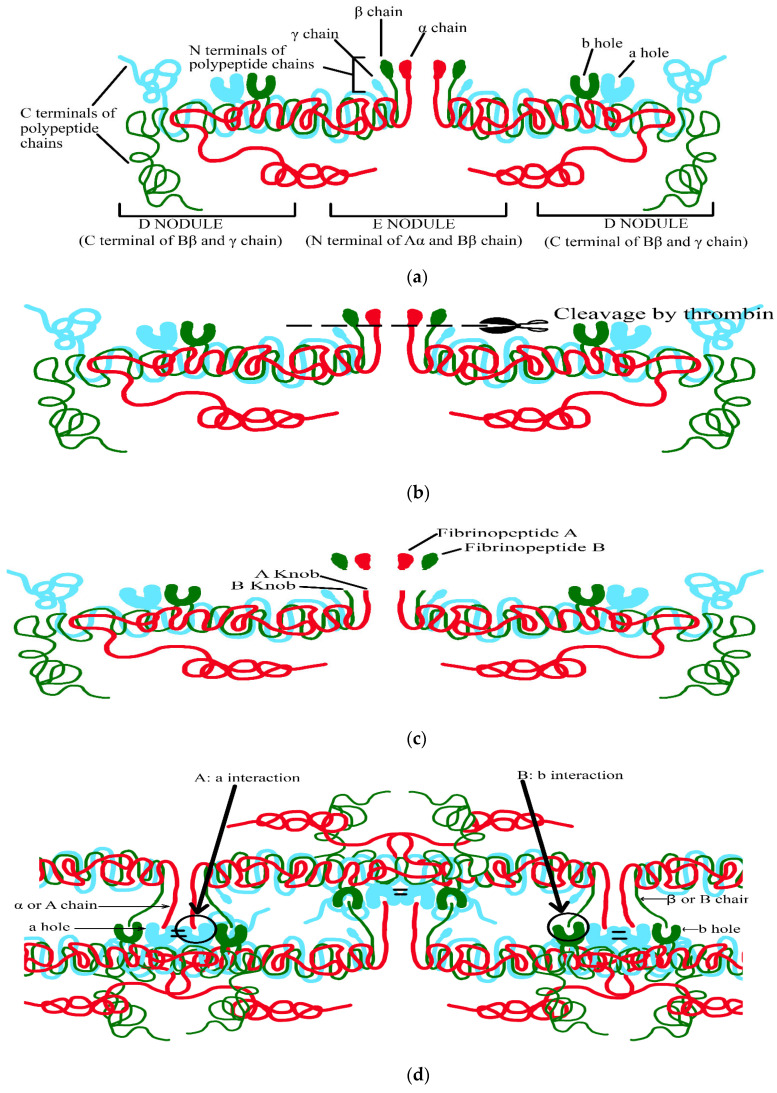

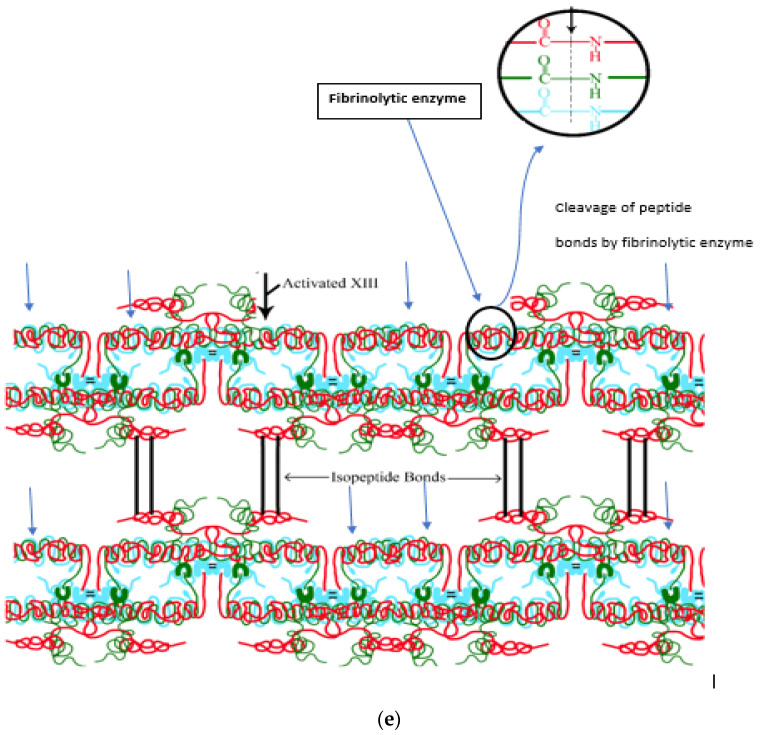
Mechanism of clot formation. (**a**) N terminal of the E nodule, C terminal of the Υ- and βB-chains, C terminal of αA-chains situated near the E nodule. (**b**) Attachment of thrombin to central E nodule cleaving N terminal peptides of Aα- and Bβ-chains. (**c**) Cleavage of Aα-chains by thrombin. (**d**) Complementary binding sites of Υ-chain and β-chain D region mediating protofibril formation and lateral aggregation of fibrinogen, respectively. (**e**) Formation of fibrin fibres.

The six polypeptide chains form:

(1) The N terminal of the E nodule,
(2) The C terminal of the Υ- and βB-chains from the D nodule facing outwards,
(3) The C terminal of αA-chains is globular and situated near the E nodule (Figure 2a) [24,25].

Assembly of these fibrinogen to form fibrin fibres is a stepwise process in which prothrombin is transformed to thrombin by the action of activated factor X and factor V, with the formation of fibrin from fibrinogen ensuing [25,26,27,28]:

(1) Thrombin attaches to central E nodule cleaving N terminal peptides of Aα- and Bβ-chains (Figure 2b).
(2) Aα-chains are firstly cleaved by thrombin at a faster rate releasing fibrinopeptide A containing N terminal (16 residues), exposing the binding site containing Gly-Pro-Arg in E region (A knob) (Figure 2c).
(3) A knob has a complementary binding site of Υ-chain D region (a hole) creating (A: a) interaction mediating the formation of protofibrils, which are metastable peptide assemblies observed during the growth of amyloid fibrils by a number of peptides (Figure 2d)
(4) Subsequently, removal of fibrinopeptide B containing N terminal (14 residues) causes a release, exposing the binding site containing Gly-His-Arg in E region (B knob) (Figure 2c).
(5) B knob also has a complementary binding site of β chain D region (b hole) creating (B: b) interaction, thus, mediating the lateral aggregation of fibrinogen (Figure 2d).

This process results in the production of protofibrils which are then converted to fibrin fibres with the help of blood clotting factor XIII [29]. 

Factor XIII is a heterologous tetramer consisting of two catalytic A subunits (XIII A) produced by bone marrow and two inhibitory/carrier B subunits (XIII B) produced by hepatocytes. By the concentrated action of thrombin and calcium ions, inactive factor XIII is transformed into active factor XIII (active transglutaminase). Thrombin first cleaves off activation peptide XIII and in the presence of calcium ions, it dissociates XIII B. This cleaved XIII A dimer is presumed to be an enzymatic active configuration of factor XIII (XIIIa) [25]. XIIIa now forms an isopeptide bond between two adjacent monomers of αα-chains and αΥ-chains forming fibrin fibres, increasing the elasticity and stabilization of the individual protofibrils and the lateral aggregate structure (Figure 2e). Activated factor XIII also incorporates α2-antiplasmin into α-chains of fibrin to prevent lysis of the blood clot [30].

Elevated levels of fibrinogen increase the fibrin network concentration, blood clot rigorousness and resistance to the fibrinolytic process increasing the risk of venous thrombosis or venous thromboembolism [31]. Fibrin polymerization is an active process that is difficult to study and is monitored by available techniques like confocal microscopy, electron microscopy, and turbidity measurements [32].

### 3.3. Fibrin Architecture

Fibrin architecture is determined by fibrinogen and thrombin concentration. Within a certain range of thrombin concentration, the porosity, fibre dimension, and gel architecture of fibrin are formed in recalcified plasma. This concentration is determined by the initial rate of fibrinogen activation [33]. Many studies certify that denser clots (more tightly packed fibres, less porosity) lyse more slowly than clots with large pores (more loosely packed fibres) [34]. The size of the fibres and the arrangement of fibrinogen have a major impact on tissue plasminogen activator binding and rate of fibrinolysis [35].

Several factors that affect the fibrin architecture are as follows:

(1) Fibrin is formed in vivo at the site of blood vessel lesion where platelets are stimulated and bind to fibrin forming powerful adhesive forces. These fibres under tension regulate clot structure, constrain fibrin fibres, and increase their density in platelet-rich areas [36].
(2) Factor XIIIa (transglutaminase) proposes Υ-glutamyllysine crosslinking between αC- and ΥC-domains of next fibrin monomers and tightens up the lateral (flanking) attachment of protofibrils. This covalent crosslinking results in a decrease in the fibrin diameter without any modification in the number of protofibrils in fibres. Thus, decreasing the vacant fluid space volume within the fibres causes a two-fold reduction in pore size [37,38].
(3) DNA and histones that are released by activated neutrophils form neutrophil extracellular traps. These have a major effect on clot lysis as they hold lysing fibrin (large fibrin degradative products) together resulting in a delay in the fibrinolytic process [39].
(4) The contractile force (induced by fibrin) of neighbouring fibres activates platelets which acts on red blood cells (RBCs) causing a change in their configuration from biconcave to polyhedral. This change induces gap-free compression of RBCs in unoccupied spaces between fibrin fibres forming a high lytic resistance structure and strong diffusion barrier [40]. These activities of RBCs increase blood viscosity, and express phosphatidylserine on their surface, which promotes fibrin deposition during venous thrombosis and reduces clot dissolution by suppressing plasmin [31].

### 3.4. Fibrinolysis

Fibrinolysis is the enzymatic breakdown of blood clots. The fibrinolytic system is composed of inactive proenzymes like plasminogen which is converted to plasmin (an active enzyme), degrading insoluble fibrin fibres into soluble FDPs (fibrin degradation products) [41].

**Figure 3:**
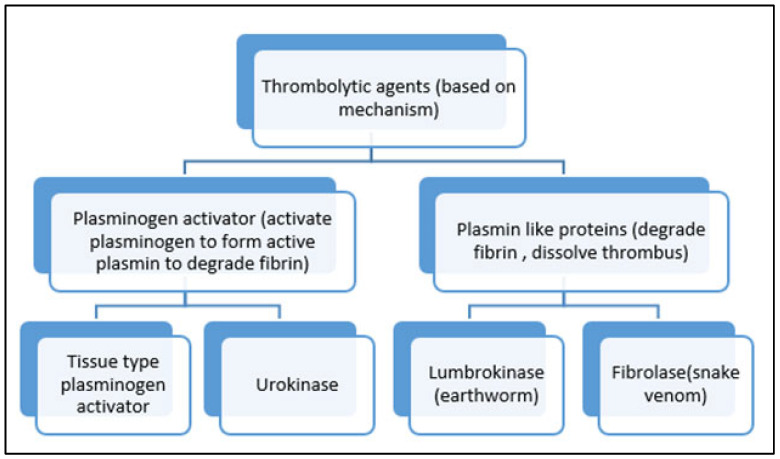
Categorization of thrombolytic agents on the basis of mechanism of action.

Fibrinolysis activation is regulated by [42]:

(1) Tissue-type plasminogen activator (t-PA) which is enhanced in the presence of fibrin.
(2) Urokinase-type plasminogen activator (u-PA), which binds to specific u-PA receptors, enhancing the activation of cell-bound plasminogen (Figure 3).

**Figure 4:**
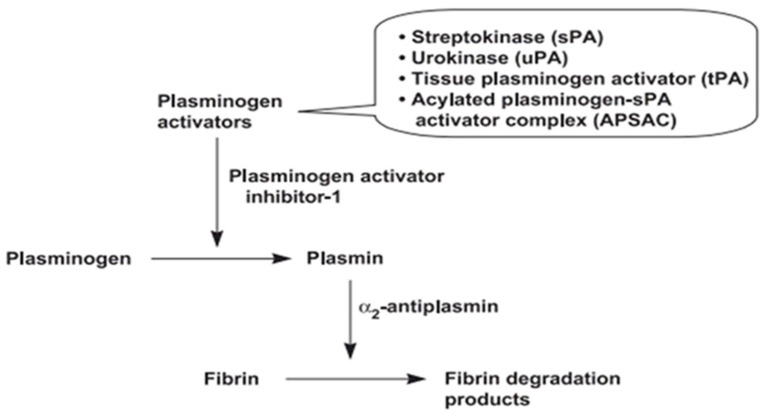
Schematic representation of fibrinolysis and proposed mode of action of fibrinolytic protease.

In fibrinolysis, fibrin plays two major roles: it enhances the production of tissue plasminogen activator, and it also acts as a substrate for plasmin (Figure 4) [43].

#### Components of the Fibrinolytic System

(1) Plasminogen

Plasminogen (single polypeptide) is a glycoprotein of 92 kDa, consisting of 791 amino acids, 24 disulfide bridges and 5 homologous kringles (a triple loop structure having lysine binding sites for fibrin) [42].

Circulated plasminogen has amino terminal glutamic acid (glu-plasminogen), which upon proteolysis is modified to amino terminal lysine plasminogen (lys-plasminogen)—hydrolysis of the Lys-77—Lys-78 peptide bond forms modified zymogen that more readily binds to fibrin. Lys-plasminogen tends to be more readily activated by tissue plasminogen activator than Glu-plasminogen and its activation rate is increased by the addition of fibrinogen [44].

The peptide bond of plasminogen is cleaved by t-PA or u-PA between Arg-561 and Val-562, forming plasmin. This plasmin contains two polypeptide chains:

- Heavy chains have an N-terminal part of plasminogen including five kringles.
- Light chains having the C-terminal part of plasminogen containing serine peptidase (catalytic triad: His-603, Asp-646, Ser-741) [38].

(2) Tissue-type plasminogen activator (t-PA).

t-PA is a proteolytic enzyme that activates plasminogen to form plasmin and is found in blood (thrombolytic agent) and the brain (promoting neuronal synaptic plasticity) [45]. T-PA may be obtained as a single polypeptide chain of molecular weight of 72 kDa. Cleavage of the t-PA peptide bond Arg-275-Ile-276 by plasmin changes t-PA to disulphide-linked, double polypeptide chains of molecular weights of 30 to 40 kDa each [46].

Five discrete structural domains are present in t-PA, encompassing finger-growth factor–kringle 1–kringle 2–protease (F-G-K1-K2-P). It comprises several potential binding sites for cells and fibrin where F and K2 are the most important sites for fibrinolysis, but mutagenic analysis specifies that K2 is less concerned with fibrin binding than anticipated [47]. These domains are responsible for attachment to fibrin, cellular receptors, and fibrin-specific plasminogen activation [42]. It also has high mannose carbohydrate on Asn-11, an O-linked α-fucose residue on Thr-61, and complex oligosaccharide on Asn-448 which regulates its binding to cell surface receptors [48]. The discharge of t-PA is regulated through a diversity of interventions like bradykinin, adrenaline, thrombin, vasopressin, histamine, gonadotropins, exercise, acetylcholine, shear stress, and venous occlusion [49].

(3) Urokinase

Prourokinase (single chain urokinase-type plasminogen activator—pro-UK) is a pioneer of two active chains, urokinase plasminogen activator (u-PA). The prourokinase can be transformed to active u-PA by the hydrolysis of the Lys-158-Ile-159 peptide bond via plasmin, factor XII, trypsin, and plasma kallikrein [50]. u-PA consists of two chains with molecular weights of 20 and 30 k Da held together by a disulfide bridge. It is a serine protease that activates plasminogen to form plasmin. Less activity than u-PA and resistance to plasmin activation is seen in the case of thromb-UK (two-chain form) which is formed by the hydrolysis of the Arg-156-Phe-157 peptide bond of pro-UK by thrombin [51].

Urokinase plasminogen activator receptors (u-PAR) are specific membrane protein receptors and are assigned as CD 87 antigens. They are a highly glycosylated protein (50–65 kDa) linked to the plasma membrane by glycosylphosphatidylinositol [52]. They are encoded by 335 amino acids of which the initial 22 amino acids comprise the signal peptide. They contain a kringle module and a serine-binding domain. They are expressed in neutrophils, activated T-cells, mononuclear phagocytes, several types of tumour cells, and endothelial cells. They have a higher affinity for u-PA converting into active form on binding, thus gaining the capability to enzymatically digest plasminogen to plasmin [53]. Pro-UK as well as u-PA can both bind to u-PAR but thrombolysis by pro-UK is more effective and specific than u-PA. u-PA has a decreased affinity for fibrin fibres than t-PA and is a dynamic plasminogen activator mutually in the presence and absence of fibrin [54,55].

(4) Plasmin

The fibrinolytic enzyme plasmin is produced from plasminogen by activators such as t-PA and u-PA. Plasmin is a serine protease that cuts fibrin chains forming solvable FDPs exposing carboxyl-terminal lysine residues. This modification of the fibrin surface structure has major implications for plasmin, plasminogen, and t-PA-possessing kringle domains which mediate binding through lysine residues, affecting the regulation of fibrinolysis. The modification on the fibrin surface increases the rate of plasmin formation by three-fold when t-PA is a plasminogen activator [56].

Other roles of plasmin are:

- Plasmin deactivates and cleaves various clotting factors FV, FVIII, FIX, and FX in vitro which plays a major role in clot formation [57].
- The two catalytic A-subunits of active clotting factor XIII are degraded endogenously by plasmin during lysis of the blood clot [58].
- Plasmin is an important matrix metalloprotease activator, enhancing the lysis effect of plasmin on surrounding tissues [59].

(5) Plasminogen activator inhibitor

Plasminogen activator inhibitor 1 (PAI-1) consists of nine α-helixes, three β-sheets, and an exposed loop containing the active site Arg-346-Met-347. Active PAI-1’s overall structure is like the structure of other inhibitory serpins [60]. It is an important physiological inhibitor that inhibits the transformation of plasminogen to plasmin by t-PA and u-PA and plays a major part in the fibrinolytic process [61]. It also inhibits the endothelial cells present in plasma which react with single polypeptide t-PA, double polypeptide t-PA, and u-PA. Strangely, it behaves as a competitive inhibitor of the binding of t-PA to fibrin. Since the inhibition constant of PAI-1 for t-PA is of the same order of magnitude as the dissociation constant of t-PA and fibrin interaction, the formation of t-PA PAI-1 complexes resulted in impaired fibrinolysis [62].

(6) α2-Antiplasmin

α2-antiplasmin (molecular weight 51 kDa) is a major inhibitor of plasmin and belongs to the Serpin family. Its synthesis takes place in the liver as an individual chain of glycoprotein comprising 464 amino acid residues (30% of circulating antiplasmin) and short chain polypeptide chain consisting of 452 amino acids (70% having higher proteolytic activity) [63]. It circulates in plasma at higher concentrations (0.9 nmol/L) with a plasma half-life of 2.4 days [49] and inhibits plasminogen activators like t-PA and u-PA [64].

α2-antiplasmin regulates fibrinolysis in three steps [60]:

- Inhibiting the adsorption of plasminogen to fibrin: the C-terminal end of α2-antiplasmin binds with a robust affinity towards the lysine binding site, where fibrin is bound non-covalently (competitive inhibition).
- Formation of a balanced inactive complex by plasmin: after the binding of α2-antiplasmin with the lysine binding site, it is quickly cleaved via plasmin at the active site releasing the peptides and forming a covalent plasmin—α2-antiplasmin complex.
- Cross-linkage via factor XIIIa: the portion of circulating α2-antiplasmin is tightly bound to fibrin via factor XIIIa, resulting in the amplified resistance of fibrin to fibrinolysis.

### 3.5. Why Measure Fibrinolysis?

Fibrinolysis under normal circumstances is a slow and natural process. Hyperfibrinolysis or enhanced fibrinolysis is life-threatening due to blood loss. Enhanced fibrinolysis has been seen in patients suffering from liver and lung disease, prostrate surgery or major trauma, cirrhosis, menorrhagia, renal failure, obstetric complications, and in some malignancies in leukaemia patients. This bleeding process starts when there is the absence of a fibrinolytic inhibitor. To combat this situation, anti-fibrinolytic lysine analogue, aprotinin, epsilon aminocaproic acid, and tranexamic acid are used.

In the case of hypofibrinolysis or impaired fibrinolysis, which may be due to environmental or heredity origin, these may be linked to thrombosis, associated with patients suffering from hyperlipidaemia, diabetes, obesity, and atherosclerosis. Various biomarkers are used that indicate reduced fibrinolysis such as elevated plasminogen activator inhibitor, α2-antiplasmin, changes in active t-PA level, and thrombin activatable fibrinolysis inhibitor. To combat this situation, anti-coagulant agents, anti-platelets drugs, and fibrinolytic enzymes are used [5].

The measurement of fibrinolytic activity is governed by various fibrinolytic assays determining hyperfibrinolysis or hypofibrinolysis conditions.

### 3.6. Fibrinolytic Activity Assay

Different methods are proposed to observe fibrinolytic action in blood and its elements [65].

(1) Fibrin plate assay

Fibrin plate assay determines fibrinolytic mediators present in the samples. It consists of two forms:

- Plasminogen-free fibrin plate (heated): This assay allows the direct activity of plasmin-like enzymes, formed from fibrinogen solution (5 mg human fibrinogen in 7 mL of 0.1 M Barbital buffer of 7.8 pH), 10 U thrombin solution and 7 mL of 10 g agarose/Liter) to be assessed in Petri plates. Then, for inactivating fibrinolytic enzymes, the plates were heated at 80 °C for 30 min. These plates were modified by means of bovine fibrinogen, calcium chloride, thrombin, and sodium chloride. The enzyme (10–30 µL) was dropped judiciously on a fibrin plate and incubated at 37 °C temperature for 3–18 h, and clear zones were obtained. A standard curve was plotted by using standard fibrinolytic enzyme (urokinase) to examine the fibrinolytic activity of an enzyme.
- Plasminogen-rich fibrin plate (non-heated): This consists of 5 U plasminogen in addition to the above fibrinolytic solution and is not heated. It is suitable for plasminogen activators [66].

The fibrin plate assay shows uncertainty in determining the accurate lysis zone and hence another method, the microtiter plate assay, was developed to overcome this problem.

(2) Fibrin microplate assay

This is a sensitive and quantitative assay to determine the fibrinolytic activity in the samples. In this assay, fibrin clots were adsorbed in 96-well microtiter plates with specific dye integration using fibrinogen. A mixture of para-nitroaniline and then thrombin were added to wells to form fibrin (overnight incubation at 37 °C). Inhibitors were removed from plasma using alcohol before being applied to the wells. Then, 20µL of test mixture was applied in triplets with 5 µL plasminogen onto the fibrin gel. For positive reference, dilutions of urokinase (standard) were used. After 6 h incubation at 37 °C, the converted substrate (lysate) was removed with rinse buffer. A total of 20 µL plasmin (1 U/mL) was added to each of the remaining blood clots. After overnight incubation at 37 °C, complete lysis was obtained and then they were rinsed with buffer and mixed well. Fibrin was photometrically determined after dissolution by plasmin at 405 nm.

The fibrinolytic activity was observed by a difference in absorption value before and after lysis of the fibrin clot. The activity was determined by using a urokinase dose–response curve based on serial dilutions of standard urokinase.

This assay is highly reliable for the assessment of the degree of clot lysis by varying concentrations of urokinase and incubation time [67].

(3) Rapid fibrin plate assay

The long incubation period (16–20 h) is the key disadvantage of fibrin plate/microplate assay and is enhanced by plasminogen enrichment. The fibrin plates were enriched with 2 U of plasminogen to form opaque plates. The fibrin clots do not lyse spontaneously and yield prominent parallel lines for streptokinase and urokinase after 3 h of incubation. Urokinase assay is more precise as compared to streptokinase because of the shallow dose–response curve [7].

(4) Euglobulin clot lysis time (ECLT)

ECLT is used to evaluate fibrinolytic activity in plasma. It implies the interaction of the activity of t-PA with plasminogen-activating inhibitor [68]. The variation in the absorbance of recalcified euglobulin fraction at different time periods indicates the fibrinolytic activity [69].

In quantitative ECLT, turbidity is measured every 30 min using a microtiter plate reader where the midpoint between minimum and maximum turbidity determines the lysis time providing reliable and reproductive data. The mathematical examination determines critical points of lysis along with the kinetics analysis of fibrinolysis. Studies project that low ECLT values are linked with elevated plasmin–antiplasmin (PAP) complexes and free tissue plasminogen activator (tPA) levels in plasma [69,70]. It is highly recommended for surgeries like cardiovascular surgery, pharmacological surgery, and liver transplantation coagulation surgery to determine atherosclerosis, hyperlipidaemic conditions, and associated diseases [68].

(5) Global fibrinolytic capacity (GFC)

Global fibrinolytic capacity (GFC) is used to evaluate the fibrinolysis in a single sample by generating D-dimers (DD) from the fibrin clot prepared with fibrinogen and thrombin-free plasminogen [68]. Silicated fibrin tablets (25 µg) were added to plasma and t-PA and incubated at 37 °C for one-hour. The aprotinin accumulation and D-dimer production were assessed for the fibrinolytic process [65]. The method is quite costly due to the reagents used and the D-dimer evaluation to estimate fibrinolysis activity. This assay is useful for diabetes (type I and II), hypothyroidism, sepsis, chronic liver and mitral valve diseases, respiratory distress, and polycystic ovary syndromes [65].

(6) Viscoelastic method

The viscoelastic method is used to analyse the effect of blood cells and platelets on clotting and fibrinolytic processes in whole blood. This process is extremely fast and plays an important role during surgical procedures allied with blood loss, traumatic injury, liver transplant, and cardiothoracic surgery [5]. Viscoelastic changes in blood can be monitored by:

- Rotational thromboelastometry (ROTEM) computes different viscoelastic parameters like clotting time, clot growth kinetics, the pace of coagulation initiation, clot strength, and dissolution [66]. The five principal assays used with the ROTEM instrument are INTEM, HEPTEM, EXTEM, FIBTEM, and APTEM assays. The INTEM test initiates clotting via the intrinsic pathway using ellagic acid, while the HEPTEM assay uses heparinase in addition to ellagic acid. EXTEM uses tissue factor to initiate the extrinsic clotting cascade whereas FIBTEM uses cytochalasin D to inhibit platelet activity and provide clot tracing that indicates the presence of fibrinogen. This test is used extensively in cardiac and liver studies to monitor fibrinogen levels. APTEM is a modified EXTEM assay that incorporates aprotinin to stabilize the clot against hyperfibrinolysis. An electrical signal from an automatic electrical transducer leads to a graphical display supervised by a computer [71,72].
- Thromboelastography (TEG) is a non-invasive test that quickly determines coagulation rate (hypo/hyper) or solidification to fibrinolysis (involving prothrombin/thrombin/fibrin), the viscoelastic properties of blood samples during clotting under low shear stress. It uses reagents different from ROTEM and involves five different parameters: reaction time, kinetics, alpha angle, maximum amplitude, and lysis at 30 min (A30/LY30) [5,72].
- Sonoclot: This assesses the change in resistivity via a small disposable plastic probe spinning vertically on a coagulating blood sample in the cuvette. Fibrin components formed on the tip/ around the probe and on the internal wall of the cuvette increase the weight of the probe leading to an upsurge in the resistivity. This increase in resistivity is sensed via electronic circuits and transformed into an output signal. The output signal describes the viscoelastic properties of the blood coagulation initiated from fibrin development, aggregation of fibrin monomers, platelet interaction, clot retraction, and lysis [73].

Chromogenic assay. The chromogenic method is based on determining the target proteolytic activity with specific chromogenic peptide substrates of plasmin (H-D-Val-Leu-Lys-pNA or similar), tissue plasminogen activator (H-D-lle-Pro-Arg-pNA or similar), and urokinase (pGlu-Gly-Arg-pNA or similar). As a rule, the sample is incubated with the substrate for 5 min at 37 °C. As a result of the reaction, a chromophore (para-nitroanaline) is split off from such substrates—para-nitroanilides—and absorption at 405 nm is measured in the mixture. The concentration of released para-nitroanaline is directly proportional to the activity of the proteolytic enzyme in the sample. The method is applicable to both mini-tubes and plates.

### 3.7. Microorganisms: Important Source of Fibrinolytic Enzymes

Numerous sources of fibrinolytic enzymes have been discovered such as microorganisms, snakes, earthworms, insects, plants, and fermented products. However, microorganisms have emerged as the most important sources, especially the genus *Bacillus* from traditional fermented foods [6]. A potent fibrinolytic enzyme (nattokinase) is used in thrombolytic therapy by cleaving the isopeptide bonds of fibrin (Figure 3) [74]. These fibrinolytic enzymes can be procured from different microbial sources such as bacterial species like *Streptococcus hemolyticus* (streptokinase) [75], *Bacillus subtilis* YF 38 (nattokinase) [8], *Staphylococcus aureus* (Staphylokinase) [76], *Bacillus* sp. DJ-4 (Subtilisin DJ4) [77], fungal species like *Arthrobotrys longa* [78], *Aspergillus oryzae* [79], *Aspergillus ochraceus* [80], *Aspergillus versicolor* [81], *Fusarium* sp. [82], *Penicillium* sp. [83], *Rhizopus chinensis* [84], *Sarocladium strictum* [85], or mushrooms such as *Cordyceps militaris* [86] and *Armillaria mella* [87].

Biochemical attributes of microbial fibrinolytic enzyme

The biochemical attributes of microbial fibrinolytic enzymes involve molecular weight, temperature optimum of activity, pH optimum of activity, substrate specificity, thermo- and pH-stability [2].

Based on catalytic properties, fibrinolytic enzymes are composed of different types of proteases. These proteases (synonyms: proteinases, peptidases, proteolytic enzymes) can hydrolyse peptide bonds in proteins. They can be found in less as well as in more complex organisms. These enzymes have excessive importance due to their major role in biochemical and cellular processes, and the life cycles of pathogens. Based on their catalytic mechanism, they are grouped as endopeptidase (cleavage takes place within the peptide backbone) and exopeptidase (cleavage takes place at the end of the peptide backbone) [88,89].

- Endopeptidase: Serine protease: trypsin, thrombin, chymotrypsin, subtilisin, etc.
  Cysteine protease: rhinovirus 3C, papain, etc.
  Metalloprotease: collagenase, thermolysin, etc.
  Aspartic protease: pepsin and cathepsin [83].

- Exopeptidase: Serine protease: carboxypeptidase Y.
  Cystine protease: cathepsin and DAPase.
  Metalloprotease: carboxypeptidase A, carboxypeptidase B [83].
  Serine and metalloprotease have catalytic properties of fibrinolytic enzymes.

*Serine protease.* Most of the members are comprised of endopeptidase which varies extensively in their specificity. The cascades of consecutive activation of serine protease are required for the initiation of blood coagulation, complement fixation, and fibrinolysis process [90]. Enzymatic active members of the chymotrypsin family include His, Asp, and Ser whereas the enzymatic active members of the subtilisin family include different orders as Asp, His, and Ser. Therefore, we can say that serine protease represents different evolutionary lines [91]. Serine protease follows two-step reactions: acylation followed by deacylation occurring via a nucleophilic attack on intermediate by water, ensuing in the hydrolysis of a peptide.

These proteases are recognized by their irreversible inhibition by tosyl-L-lysine chloromethyl ketone (TLCK), L-3-carboxytrans 2,3-epoxypropyl-leucylamido (4-guanidine) butane, 3,4-dichloroisocoumarin (3,4-DCI), diisopropylfluorophosphate (DFP), and phenylmethylsulphonyl fluoride (PMSF) [92].

Fibrinolytic enzymes associated with the serine protease family belong to *Bacillus* sp. (subtilisin) and are active at alkaline to neutral pH (optimum pH 8–10), isoelectric points about 8 and molecular weights are between 27.7 and 44 KDa. The optimum temperature has a range between 30 and 70 °C, mostly 50 °C. It contains a catalytic triad containing Ser 221, His 64, and Asp 32 without any intramolecular disulphide linkage. Examples are Subtilisin DJ 4, CFR 15 protease, Subtilisin DFE, BAFFI, etc. (Table 1) [65].

*Metalloprotease.* Metalloproteases are synthesized as inactive zymogens by heterotrophic bacteria [93]. All metalloproteases comprise one or two zinc ions and some enzymes contain one or two manganese or cobalt ions [94]. In the metalloprotease family, 13 members contain the HEXXH sequence providing two of three ligands for zinc atoms. This sequence occurs as a consensus of nine residues bXHEbbHbc (b: uncharged residue, X: any amino acid, and c: hydrophobic residue). The third ligand of the zinc atom is histidine in the case of autolysin, astacin, interstitial collagenase, and glutamic acid in the case of thermolysin, neprilysin, and alanyl aminopeptidase [91]. They can be deactivated by the addition of chelating mediators or dialysis [92].

Fibrinolytic enzymes associated with the metalloprotease family require divalent ions for their actions. They have an optimum pH between 6 and 7. Examples of metalloprotease are Ca^2+^ and Mg^2+^ for AMMP, Zn^2+^ for jeot gal and Co^2+^ Hg^2+^ for *Bacillus* sp. KDO 13 [65], as shown in Table 1.

From Table 1, common antithrombotic enzymes used for the treatment of various cardiovascular diseases are nattokinase, streptokinase, staphylokinase, serrapeptase and longolytin.

### 3.8. Nattokinase (NK)

**Figure 5:**
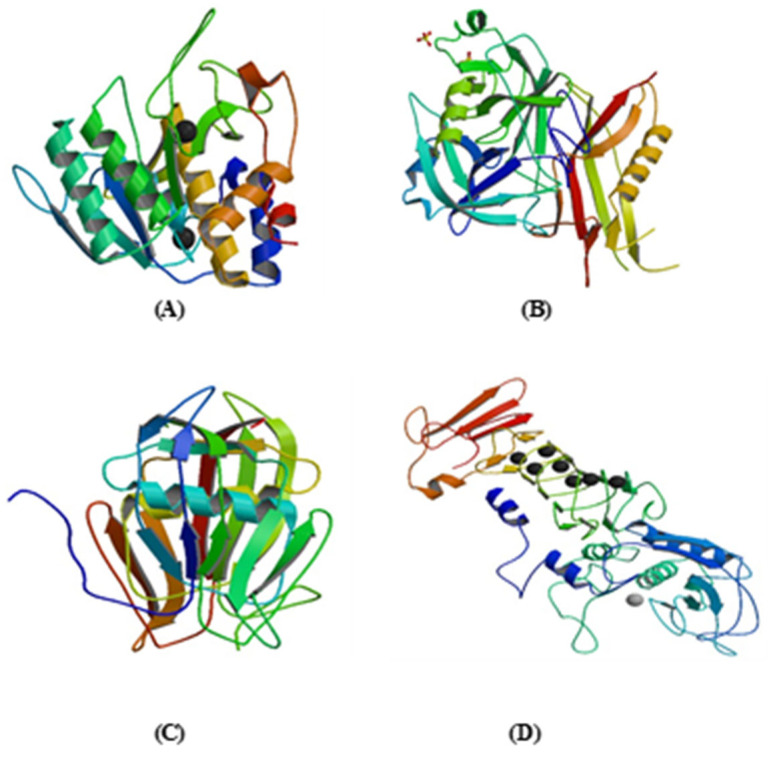
Three-dimensional structures of anti-thrombotics. (**A**) Structure of nattokinase. (**B**) Structure of streptokinase. (**C**) Structure of staphylokinase. (**D**) Structure of serrapeptase.

A Japanese researcher, Hiroyuki Sumi (Chicago University Medical School) in 1980 invented natto which dissolves artificial fibrin. Sumi and his team members obtained an enzyme from natto that not only degrades fibrin clots but also degrades plasmin substrate. He termed this fibrinolytic enzyme “nattokinase”. Natto is a fermented cheese-like food that has been used in Japan over thousands of years. It is made up of soybeans which are cooked and fermented with the action of bacterium *Bacillus subtilis* [129,130]. It degenerates fibrin directly during clot lysis with an action like plasmin and indirectly by affecting plasminogen activator activity (Figure 5A) [130,131].

Nattokinase (NK) is a serine protease with 275 amino acid residues, and its molecular weight is 27.728 Da. It has immense homology with subtilisin, and DNA sequencing displays 99.5% homology with subtilisin E and 99.3% homology with *B. amylosacchariticus*.

NK’s effects are as well-known as aspirin (a well-known blood thinner) where NK improves blood flow with no side effects as compared to aspirin which often triggers gastric ulcers and bleeding. It is resistant to the acidic pH of the stomach (absorbed in the digestive tract), has a high pH (10), and resists high temperatures such as 50 °C [130]. It has various other applications such as a functional food additive, reduces blood viscosity and plasma II concentration, etc. [132].

In human trials, patients undergoing dialysis, patients with cardiovascular diseases, and healthy volunteers were administered orally with two capsules of (2000 FU/capsule) daily. After two months, it was observed that there was a decrease in factors VII and VIII due to which fibrinogen was observed in all three cases, causing no side effects with stable heart rate, uric acid production, and body weight. When dogs were administered orally with four NK capsules (2000 FU/capsule), the major leg vein containing chemically induced blot clot (thrombi) was dissolved completely in 5 h, restoring normal blood flow. Similarly, in the case of a rat’s thrombosis in the carotid artery, this was treated with NK, and 62% of arterial blood flow was recovered. NK improves various diseases like atherosclerosis, Alzheimer’s disease, hypertension, and stroke and is commercially used in the United States, Korea, Japan, Canada, China, and European Union Countries [129].

C. Yongjun et al. performed an experiment to improve the activity of nattokinase. Three homological genes from *B. natto* AS 1.107, *Bacillus licheniformis* CICC 10092, and *Bacillus amyloliquefaciens* CICC 20164 were intermixed properly to yield a mutant library. After the three cycles of DNA shuffling, one desired mutant of 16 amino acids was attained. For screening, the mutant library for improved activity, the plate-based method was used. The three-dimensional structure was obtained based on parental NK. The hydrophobic pocket present at the active site was broadened due to amino acid substitutions and this may lead to change in catalytic as well as enzymatic activity. The catalytic activity of mutant NK was found to be 2.3 times higher than the wild-type nattokinase [133].

### 3.9. Streptokinase (SK)

Dr. William Smith Tillet with Sol Sherry (scholar), in 1933, serendipitously laid the basis for the usage of streptokinase (SK) as a thrombolytic mediator in the treatment of acute myocardial infarction [134].

SK is an active plasminogen activator consisting of 414 amino acid residues. It forms a 1:1 stochiometric complex with plasmin (activating plasminogen to plasmin), thus resulting in clot lysis by the proteolytic cleavage of fibrin. It is also included in the World Health Organization’s Model List of Essential Medicines and is clinically used an intravenous thrombolytic agent for preventing cardiovascular diseases [118].

Streptokinase N-terminal domain has low activation ability to complement plasminogen (60–414 amino acids) and C-terminal used in plasminogen substrate recognition and stimulation. Initially, 59 amino acid residues appear to have numerous binding domains for plasminogen. The important binding sites that are present in streptokinase are Asp41 and His48 and the coiled region of the Υ-domain plays an important role in plasminogen activation. Its mechanism is fibrin-dependent and independent (Figure 5B) [75,135]. As streptokinase is a non-human protein, its entry into the circulatory system may cause different anaphylactic reactions, including death, depending upon the concentration of anti-streptokinase antibodies existing in the blood circulation.

It is degraded by plasmin at proteolytic sites Lys59 and Lys386 due to which it has a shorter half-life; hence, modifications are performed to enhance its half-life by recombinant DNA technology, chemical or enzymatic alteration of indigenous streptokinase, and by genetic mutation. Recombinant streptokinase produced by yeast *Pichia pastoris* is resistant to proteolytic cleavage by plasmin [75].

### 3.10. Staphylokinase (SAK)

Staphylokinase is produced by lysogenic strains of *Staphylococcus aureus,* a subsidiary activator of plasminogen, and is a part of third-generation fibrinolytic enzymes [59,136]. It forms a 1:1 stochiometric complex with human plasmin catalysing further stimulation of plasminogen. This complex is sensitive to accelerated inhibition by α2-antiplasmin unless it is bound to fibrin by lysine-binding sites [137].

It is a 15.5 kDa protein consisting of 136 amino acids (single chain without disulfide linkage). Its 3D crystal structure involves five stand beta-sheets, a central alpha-helix (Lys-57-Thr-71) connected together by loops (Figure 5C) [55,138]. The main interaction between staphylokinase and plasminogen or staphylokinase and other staphylokinases (during the dimerization process) occurs at the region possessing alpha helix. The mutation in the active region (central alpha helix) results in a complete loss of activity of plasminogen. The N-terminal part (Lys 10) is responsible for proteolytic hydrolysis and protein interaction, whereas the C-terminal part (Lys-135-Lys-136) and its sterically positioned flexible loop (Lys-54) is responsible for dimerization and determinant of staphylokinase–plasminogen interaction [59].

Another role of SAK is its ability to nullify the activity of host antimicrobial peptides constituting various peptides secreted by mammals and other organisms. Hence, it plays its role against invading pathogens through a defence mechanism. SAK is able to bind with α2- antiplasmin that are secreted by human neutrophil proteins (HNPs) forming the SAK-HNP complex. This complex results in the mutual inhibition of protein bactericidal activities and thrombolytic activities [139].

### 3.11. Serrapeptase (SRP)

Serrapeptase is a proteolytic enzyme (metalloprotease with three zinc atoms and one active site) with molecular weight ranges from 45 to 60 kDa catalysing hydrolysis of peptide bonds in peptides. It is also known as serratiopeptidase, or serratia peptidase (SRP), due to its origin in *Serratia marcescens.* It has an affinity towards dead threads of proteins present in silkworms and dissolves them to make cocoons; it also dissolves non-living tissues present in mammals including plaques, mucous, and blood clots. It also degrades various protease inhibitors of the immune system and is the main reason for infection in mammalian epithelial cells [95].

It consists of 470 amino acids lacking sulphur-containing amino acids such as methionine and cysteine (Figure 5D) [90,140]. The activity of the serrapeptase enzyme is enhanced by zinc atoms [90]. It contains a zinc-binding consensus (HEXXHXXGXXH) where three histidine residues are zinc ligands, and the catalytic base is glutamic acid [140,141]. This enzyme belongs to the Serralysin group of enzymes which cleaves peptide bond linkage between Asn-Gln, Tyr-Tyr, Arg-Gly, CysSO_3_H-Gly, Tyr-Tyr, His-Leu, Gly-Ala, Try-Thr, Gly-Gly, Phe-Tyr Ala-Leu, and Tyr-Leu; hence, it has broad substrate specificity [142].

Expression of SRP is available in the form of enteric-coated tablets (dry-coated with enteric polymer) as it undergoes gastric hydrolysis at a low pH decreasing the stability in the gastrointestinal tract. Hence, to maintain the stability of SRP, the recommended doses are 5–10 mg tablets three times a day. Orally given serratiopeptidase alters the viscoelasticity of sputum in patients (chronic airway disease), it also has various other side effects such as GI disturbance, nausea, and anorexia. Topical application of the SRP enzyme in the form of ointments and gels at the site of action increases the potential activity of the enzyme in the treatment of local inflammatory reactions. Non-steroidal anti-inflammatory drugs (NSAIDs), such as diclofenac sodium, ketoprofen, etc., which are used to treat chronic and acute arthritic conditions cause gastric irritation (causing ulcers) and other side effects. Hence, anti-inflammatory properties of SRP could be used as a suitable alternative to NSAIDs. It also enhances the efficiency of some antibiotics such as ampicillin, cefotiam, ciclacillin, cephalexin, and minocycline. A team of Italian researchers suggested that SRP enhances the effectiveness against microbial biofilms and inhibits its formation [143].

### 3.12. Longolytin

Longolytin is the preparation of extracellular proteolytic enzymes obtained from the culture fluid of the strain *Arthrobortys longa* Mecht. No. 1. The drug has fibrinolytic, thrombolytic, esterase, small proteolytic, and plasminogen activator activities. When separating longolitin, six protein fractions were identified, among which only one was fibrinolytically active. The enzyme had a pI of 3.7 and a molecular weight of 28.6 kDa. The optimal activity was at pH 6.0–9.0 and a temperature of 37 °C. Based on the action of inhibitors, the enzyme was tentatively assigned to serine-type proteinases containing thiol groups. The effectiveness of longolytin has been proven in in vitro and in vivo experiments. A pronounced affinity of the drug for fibrin was shown, and its intravenous administration to animals increased the fibrinolytic and activator properties of plasma and proved its local effect on the structure of blood clots. An increase in the amount of plasmin in the blood of animals that received high doses of the drug showed the ability of the drug to activate plasminogen. Longolytin was first proposed as a thrombolytic drug for the treatment of thrombophlebitis and phlebothrombosis, due to its ability to penetrate the epidermis and underlying soft tissues into the microcirculation system and systemic circulation and cause adequate physiological and biochemical reactions.

A model of venous thrombosis on the rabbit marginal ear vein was developed by Podorol′skaya et al., 2007 [127]. The thrombolytic activity of longolytin applied externally onto the thrombotic venous segment was evaluated. When applied externally, longolytin (both individually and in combination with heparin) causes a significant acceleration of thrombolysis, acting locally on the thrombus structure, and does not affect haemostasis. Heparin significantly accelerated the process of dissolution of blood clots only when it was used together with longolytin. Thus, longolytin reduced the time of thrombus dissolution by 2 times and increased the rate of thrombolysis by 4.5 times. The combined use of longolytin and heparin increased jugular vein thrombolysis by 30 times compared with the control group. Biochemical indicators of haemostasis (fibrinogen content, fibrinolytic activity, recalcification time) remained unchanged during thrombolysis both in the experiment and in the control, which indicates the specificity and selectivity of longolytin. The introduction of longolytin into the stomach cavity and into the oral cavity of rats also demonstrated the effect of a significant increase in the fibrinolytic and anticoagulant activity of the blood of animals. Sharkova and Podorolskaya, 2017 [128] have used longolytin by per os administration in rats to reveal the influence on haemostasis and fibrinolysis. In their experiments, 120 white rats were introduced to a 0.1 mL 3% solution of longolytin in glycerol (activity 30–40 C.U.) orally every day for 7 days and observed an increase in anticoagulant and fibrinolytic activities in experimental rats. The resulting effects turned out to be prolonged, remaining for another week after the drug was discontinued and creating a favourable antithrombotic background in the animal body, in contrast to the inhibition of fibrinolysis characteristic of intravenous administration at the end of the course of use. So, longolytin can be used orally for both therapeutic and prophylactic purposes [144].

### 3.13. Clinical Significance of Fibrinolytic Enzymes

Fibrinolytic enzymes are clinically administered for the treatment of myocardial infarctions, strokes, cardiac and respiratory failure. Therefore, in this section, we intend to discuss some of the clinical trials of fibrinolytic enzymes stating their significance and importance.

The efficacy of a single dose of nattokinase (2000 FU) was evaluated in a double-blind, placebo-controlled cross-over nattokinase intervention human trial. The results showed that after the administration of nattokinase, D-dimer concentrations at 6 and 8 h and fibrin/fibrinogen degradation at 4 h elevated significantly with respective *p*-values of < 0.05. Also, antithrombin concentration in the blood was also higher at 2 and 4 h, and thus the outcomes suggest that fibrinolysis and anti-coagulation are enhanced using nattokinase via numerous pathways [145]. In addition, treatment with streptokinase in a controlled clinical trial revealed that angiographic evidence of thrombolysis was significantly greater (*p* < 0.01) in patients treated with streptokinase when compared to heparin. However, after-effects such as bleeding were more common with streptokinase than with heparin but was not a critical concern [146]. Another clinical trial by Gusev EI et al. performed a multicentre, open-label, randomized, parallel-group, and non-inferiority trial and studied the effect of non-immunogenic recombinant staphylokinase versus alteplase for patients with acute ischaemic stroke 4–5 h after symptom onset. The difference in the rate of favourable response at day 90 was 9.5% (95% CI −1.7 to 20.7) and the lower limit did not cross the margin of non-inferiority (*p* non-inferiority <0.0001). After-effects such as symptomatic intracranial haemorrhage affected 8% and 3% of patients (*p* = 0.087) in the non-immunogenic staphylokinase group and alteplase group, respectively. Also, on the follow-up day 90, 14% and 10% of patients (*p* = 0.32) died in the non-immunogenic staphylokinase group and alteplase group, respectively [147]. These brief data from clinical trials indicate that fibrinolytic enzymes might serve as an efficient alternative to synthetic antithrombotic agents. However, treatments with these drugs are associated with an increased risk of complications such as haemorrhage, and therefore, the search for safer and more efficient methods is evident.

### 3.14. Other Potential Applications of Fibrinolytic Enzymes

Fibrinolytic enzymes along with blood clot dissolution, exhibit numerous other potential applications in food, industrial, and clinical sectors. They have been reported as aiding in blood pressure regulation and proteolysis in addition to fibrin. Fibrinolytic enzymes have also found their applicability as antimicrobials, detergent additives, anti-inflammatory agents, etc. [2]. A randomized clinical trial reported the positive effects of nattokinase on blood pressure/hypertension and confirmed that nattokinase results in a reduction in systolic and diastolic blood pressure [148]. Another study detailed that oral administration of nattokinase and serrapeptase is effective against Alzheimer’s disease [149]. Serrapeptase exhibits significant anti-inflammatory and other essential applications along with anti-thrombotic activity [6,66,150,151]. In addition, serrapeptase has shown a potent effect with respect to venous inflammatory diseases and chronic airway diseases such as decreased neutrophil count, sputum output and viscosity, and chronic sinusitis [152,153,154,155]. Scientific studies also provide insight into the significant role of fibrinolytic enzymes in food fortification and nutraceutical applications [156,157]. Fibrinolytic protease from *Lactobacillus plantarum* KSK-II was found to inhibit the growth of *S. aureus* (29%), *B. cereus* (21%), *P. aeruginosa* (13%), *P. vulgaris* (10%), and *E. coli* (7%) and thus exhibit anti-microbial activity [6]. 

Fibrinolytic enzymes also aid in proteolysis in addition to fibrin and are considered as an apt detergent additive. The enzyme from *L. plantarum* was found to hydrolyse plasma proteins along with collagen and fibrin and was also compatible/stable with the detergent formulations of Persil (112%), X-tra^®^ (98%), Ariel^®^ (92%), Tide^®^ (86%), Lang^®^ (81%), Dac^®^ (80%), Isis^®^ (77%), Bonux^®^ (75%), Dixan^®^ (67%), and Oxi^®^ (64%) [6]. A detergent-resistant nattokinase from *B. subtilis* showed an increase of 141% with non-ionic detergents (Tween-20, Tween-80, and Triton X-100) [158]. Furthermore, an enzyme from *Bacillus* sp. IND12 bovine serum albumin, chicken skin, hydrolysed egg white, and goat blood clots was recommended for use in both wastewater treatment and clinical practices [159].

## 4. Conclusions

In the present review, we have discussed in detail the mechanism of blood clotting, different microbial sources used as anti-thrombotic agents, and their mechanism of action. We have summarized the mechanism of blood clot formation, and clot lysis (fibrinolysis) with the help of plasminogen activators or plasmin-like molecules involving fibrinolytic enzymes. Different methods are proposed to observe fibrinolytic action in blood and its elements. Fibrinolytic activities can be linked with the patient’s cardiovascular disease status which can be taken care of well in advance to avoid serious cardiac arrest. Fibrinolytic enzymes have the capability to dissolve blood clots. These enzymes obtained from microorganisms cleave fibrin fibres and clot peptide bonds of chains (αα-, ββ-, Υ-) forming fibrin degradative products, dissolving the blood clots present in blood vessels for normal blood flow. Several types of reported/ commercially available fibrinolytic enzymes have also been described along with their clinical trials and applications. Human trials suggest microbial fibrinolytic enzymes as efficient substitutes to other antithrombotic agents, but after-effects such as hemorrhage leave an urgent urge to search for more safer and potent therapies. Lastly, other potential applications of fibrinolytic enzymes such as detergent additives, blood pressure regulators, anti-microbial/anti-inflammatory agents, etc., pave the way for their diverse use. However, the mechanisms behind the above-mentioned uses are uncertain so far.

## Figures and Tables

**Table 1:** Microbial fibrinolytic enzymes and their properties.

Fibrinolytic Enzymes	Micro-Organisms Associated	Sources for Production	Physicochemical Properties	Functional Moiety	Mechanism of Action	References
Streptokinase	*Streptococcus hemolyticus*	Exudates of infected wounds	47 kDa pH 7.5 37 °C	Single polypeptide chain (414 amino acids) with multiple structural domains (α, β, Υ)	plasminogen activation for the formation of β-domain SK plasminogen complex	[75,93]
Staphylokinase	*Staphylococcus aureus*	Human skin	15.5 kDa pH 8.5 37 °C	Single polypeptide chain (136 amino acids) without disulphide bridge	plasminogen activation due to higher affinity with plasmin	[76,94]
Serrapeptase	*Serratia marcescens* E 15	Intestine of silkworm	45–60 kDa pH 9.0 40 °C	Metalloprotease with one active site and three Zn atoms	Cleavage of peptide bond linkages	[95]
Nattokinase (wild Type)	*Bacillus subtilis* YF 38, natto	Fermented soybean Natto	27.7 kDa pH 8.6	Presence of catalytic triad (Asp-32, His-64 and Ser-221) and one oxyanion hole (Asn-155)	Resemblance with plasmin and enhanced production of plasmin and clot dissolving agents	[8,96]
Nattokinase	*Pseudomonas aeruginosa* CMSS (new strain)	Cow milk	21 kDa pH 7.0 25 °C	Like wild-type nattokinase with a two-fold increase in enzyme activation	Same as wild type nattokinase	[97]
CK fibrinolytic enzyme	*Bacillus* sp. CK	Chungkook-jang (Korea)	28.2 kDa pH 10.5 70 °C	Thermolytic alkaline serine protease (1882 protein atoms, 2 Ca^2+^ ions, and 44 water molecules)	Higher tissue plasminogen activator production.	[98,99]
Fibrinolytic enzyme	*Bacillus* sp. KA38	Jeot-gal (fermented fish, Korea)	41 kDa pH 7.0 40 °C	Metalloprotease	Degrade fibrin or form plasmin from plasminogen	[100]
CFR 15 protease	*Bacillus amyloliquefaciens* MCC2606 (strain CFR 15)	Dosa batter	32 kDa pH 10.5 45 °C	Serine protease with a catalytic triad (His-57, Ser-195, Asp-102)	Hydrolysis of αα-, ββ-, ϓ- chains of fibrin	[101]
*B*. *amyloliquefaciens* An6 fibrinase (BAF1)	*Bacillus amyloliquefaciens* An6	*Mirabilis jalapa* tuber powder (MJTP)	30 kDa pH 9.0 60 °C	Serine protease	Degrade fibrin or form plasmin from plasminogen	[102]
Subtilisin DJ-4	*Bacillus* sp. DJ -4	Doen-jang, Korea	29 kDa pH 10.0 40 °C	Plasmin-like serine protease	Rapid hydrolysis of αα-, ββ-, ϓ- chains of fibrin	[77]
Subtilisin QK02	*Bacillus* sp. QK02	Fermented soybean	28 kDa pH 8.5 55 °C	Serine protease with a catalytic triad (Asp-32, His-64 and Ser-221)	Cleaves peptide bond linkages	[103]
Subtilisin DFE	*Bacillus amyloliquefaciens* DC 4	Douchi (China)	28 kDa pH 9.0 48 °C	Serine protease	High specificity towards fibrin and hydrolyses thrombin	[104]
Fibrinolytic enzyme	*Bacillus tequilensis* CWD-67	Dumping soil	22 kDa pH 8.0 45 °C	Chymotrypsin-like serine metalloprotease containing hydrophobic S1 pocket	Hydrolysis of αα-, ββ-, ϓ- chains of fibrin	[105]
BacillokinaseII	*Bacillus subtilis* A1	Local soil (Korea)	31.4 kDa pH 7.0 50 °C	Chymotrypsin-like serine protease	Degrade fibrin and act as plasminogen activator	[106]
Fibrinolytic enzyme	*Bacillus* sp. KDO- 13	Soybean paste (Korea)	45 kDa pH 7 60 °C	Metalloprotease with Catalytic domain with 170 amino acids, hinge region, and hemopexin domain of 200 amino acids	Degrade fibrin or form plasmin from plasminogen	[107,108]
Fibrinolytic enzyme	*Bacillus thuringiensis* IND 7	Cow dung	32 kDa pH 9.0	Serine protease	Degrade fibrin or form plasmin from plasminogen	[109]
Bafibrinase	*Bacillus* Sp. AS-S20-I	Soil (Assam)	32.3 kDa 7.4 pH 37 °C	Catalytic triad (Ser-221, His-64 and Asp-32) without intramolecular sulphide bond	Cleaves chains of fibrin (α, β) and fibrinogen	[110]
Subtilisin BK 17	*Bacillus subtilis* BK17	Decaying rice plant (Korea)	31 kDa	Serine protease	Degrade fibrin or form plasmin from plasminogen	[111]
Fibrinolytic enzyme	*Bacillus subtilis* KCK-7	Chungkookjang (fermented food)	45 kDa pH 7.0 60 °C	Serine protease requires hydroxyl group for activity	Degrade fibrin or form plasmin from plasminogen	[112]
Douchi fibrinolytic enzyme	*Bacillus subtilis* LD 8547	Soybean fermented food (China)	30 kDa	Serine protease	Activate t-PA	[113]
Fibrinolytic enzyme	*Paenibacillus* sp. IND8	Cooked Indian rice	-	-	Degrade fibrin or form plasmin from plasminogen	[114]
SW 1	*Streptomyces* sp. Y405	Soil isolate	30 kDa pH 8.0	Serine protease and metalloprotease	Degrade fibrin or form plasmin from plasminogen	[115]
Fibrinolytic enzyme	*Streptomyces rubiginosus*	Marine soil	45 kDa pH 7.2 32 °C	-	Degrade fibrin or form plasmin from plasminogen	[116]
Fibrinolytic enzyme	*Streptomyces* sp. MCMB-379	Seed culture	-	Serine endopeptidase type	Cleaves fibrin fibres by degradation of chains	[117]
β Haemolytic Streptokinase	*Streptococcus equinus*	Bovine milk	-	-	Degrade fibrin or form plasmin from plasminogen	[118]
Fibrinolytic enzyme	*Bacillus cereus* SRM-001	Chicken dump yard	28 kDa pH 7.0 37 °C	Serine protease	Plasmin catalysed hydrolysis of fibrin	[119]
Fibrinolytic enzyme	*Bacillus cereus* IND 5	Cuttle fish waste and cow dung	47 kDa pH 8.0 50 °C	Serine protease	Degrade fibrin or form plasmin from plasminogen	[120]
Fibrinolytic enzyme	*Bacillus pumilus*	Gembus (Indonesia fermented food)	20 kDa 50 °C	Serine protease	Degrade α- and β-chains of fibrinogen but not Υ-chain	[121]
Fibrinolytic enzyme	*Serratia* sp. KG 2–1	Garbage dump yard	pH 8.0 40 °C	Metalloprotease	Degrade fibrin or form plasmin from plasminogen	[122]
Fibrinolytic enzyme	*Shewanella * sp. IND20	Fish *Sardinella* *longiceps*	55.5 kDa pH 8.0 50 °C	Serine protease	Direct clot lysis and plasminogen activation activity	[123]
Fibrinolytic enzyme	*Cordyceps militaris*	Mushroom	28 kDa pH 7.2 37 °C	Serine protease	Activate plasminogen to plasmin	[78]
Fibrinolytic enzyme	*Lasiodiplodia pseudotheobromae*	*Aegle Marmelos* (Golden apple)	80 kDa	-	Degrade fibrin or form plasmin from plasminogen	[124]
AMMP	*Armillaria mella*	Mushroom (Korea)	21 kDa pH 6.0 33 °C	Chymotrypsin like metalloprotease	Hydrolyse α-α fibrinogen	[79]
Fibrinolytic enzyme	*Mucor subtilissimus* UCP 1262	Soil (Brazil)	20 kDa pH 9.0 40 °C	Chymotrypsin like serine protease	Properties resemble to plasmin	[125]
Fibrinolytic enzyme	*Cochliobolus lunatus*	Surface culture	pH 6.8 40 °C	-	Degrade fibrin or form plasmin from plasminogen	[126]
Longolytin	*Arthrobotrys longa*	Soil, contains nematodes	28.6 kDa pH 6.0–9.0	Serine protease contains thiol groups	Hydrolyse fibrin and activate plasminogen like urokinase	[127,128]
Fibrinolytic enzyme	*Aspergillus ochraceus* L-1	Soil	36 kDa pH 10.0–11.0 45 °C	Serine protease	Hydrolyse fibrin and fibrinogen	[79]
Fibrinolytic enzyme	*Aspergillus oryzae* KSK-3	Commercial rice-koji for miso brewing	30 kDa pH 6.0 50 °C	Serine protease	Hydrolyse fibrin and fibrinogen	[80]
Versiase	*Aspergillus versicolor*	Marine sponge *Callyspongia* sp.	37.3 kDa pH 5.0 40 °C	Metalloprotease	Hydrolyse fibrin directly and indirectly via the activation of plasminogen, and it can hydrolyse α-, β- and γ-chains of fibrinogen.	[81]
Fu-P	*Fusarium* sp. CPCC480097	Shanghai Health Creation Center of Biopharmaceutical R&D	28 kDa pH 8.5 45 °C	Serine protease	Hydrolyse fibrin and fibrinogen	[82]
Fibrinolytic enzyme	*Paecilomyces tenuipes*	Culture Collection of DNA Bank of Mushrooms, Incheon, Republic of Korea.	14 kDa pH 5.0 35 °C	Serine protease	Hydrolyse the Aα chain of human fibrinogen, but do not hydrolyse the Bβ or γ chains	[83]
Fibrinolytic enzyme	*Rhizopus chinensis* 12	Brewing rice wine	18.0 kDa pH 10.5 45 °C	Serine protease. The first 12 amino acids of the N-terminal sequence of the enzyme were S-V-S-E-I-Q-L-M-H-N-L-G and had no homology with that of other fibrinolytic enzyme from other microorganism.	Hydrolyse fibrin and α-, β- and γ-chains of fibrinogen	[84]
Fibrinolytic enzyme	*Sarocladium strictum* 1	*Arhtrobotrys longa* co-culture	35.0 kDa pH 9.0 37 °C	Serine protease	Hydrolyse fibrin and activate plasminogen like urokinase	[85]

## Data Availability

Not Applicable.
